# Transcatheter Aortic Valve Implantation and Cardiac Conduction Abnormalities: Prevalence, Risk Factors and Management

**DOI:** 10.3390/jcm12186056

**Published:** 2023-09-19

**Authors:** Michał Szotek, Łukasz Drużbicki, Karol Sabatowski, Gisella R. Amoroso, Koen De Schouwer, Paweł T. Matusik

**Affiliations:** 1 Department of Electrocardiology, The John Paul II Hospital, 80 Prądnicka St., 31-202 Kraków, Poland; 2 Department of Cardiovascular Surgery and Transplantology, The John Paul II Hospital, 80 Prądnicka St., 31-202 Kraków, Poland; 3 Department of Cardiology and Cardiovascular Interventions, University Hospital, 2 Jakubowskiego St., 30-688 Kraków, Poland; 4 Department of Cardiovascular Medicine, “SS Annunziata” Hospital, ASL CN1-Savigliano, Via Ospedali 9, 12038 Savigliano, Italy; 5 Department of Cardiology, Cardiovascular Center, Onze-Lieve-Vrouwziekenhuis Hospital, Moorselbaan 164, 9300 Aalst, Belgium; 6 Institute of Cardiology, Faculty of Medicine, Jagiellonian University Medical College, 80 Prądnicka St., 31-202 Kraków, Poland

**Keywords:** transcatheter aortic valve implantation, TAVR, conduction abnormalities, atrioventricular block, left bundle branch block, cardiovascular implantable electronic devices, pacemaker implantation, arrhythmia

## Abstract

Over the last decades, transcatheter aortic valve implantation (TAVI) or replacement (TAVR) has become a potential, widely accepted, and effective method of treating aortic stenosis in patients at moderate and high surgical risk and those disqualified from surgery. The method evolved what translates into a noticeable decrease in the incidence of complications and more beneficial clinical outcomes. However, the incidence of conduction abnormalities related to TAVI, including left bundle branch block and complete or second-degree atrioventricular block (AVB), remains high. The occurrence of AVB requiring permanent pacemaker implantation is associated with a worse prognosis in this group of patients. The identification of risk factors for conduction disturbances requiring pacemaker placement and the assessment of their relation to pacing dependence may help to develop methods of optimal care, including preventive measures, for patients undergoing TAVI. This approach is crucial given the emerging evidence of no worse outcomes for intermediate and low-risk patients undergoing TAVI in comparison to surgical aortic valve replacement. This paper comprehensively discusses the mechanisms, risk factors, and consequences of conduction abnormalities and arrhythmias, including AVB, atrial fibrillation, and ventricular arrhythmias associated with aortic stenosis and TAVI, as well as provides insights into optimized patient care, along with the potential of conduction system pacing and cardiac resynchronization therapy, to minimize the risk of unfavorable clinical outcomes.

## 1. Introduction

The incidence of aortic stenosis (AS) increases with age, affecting up to 10% of the population by the eighth decade [1]. It is the most common primary valve lesion requiring surgery or transcatheter intervention in Europe and North America, and its prevalence is rising rapidly as a consequence of the ageing population. Symptomatic severe AS has a dismal prognosis and early intervention is generally recommended. A broad spectrum of patients requires different approaches to treatment. Transcatheter aortic valve implantation (TAVI) or replacement (TAVR) was performed for the first time in 2002 [1,2]. Initially, it was considered as an alternative to surgical aortic valve replacement (SAVR) for severe, symptomatic AS in patients who had a high cardiothoracic surgery risk at clinical evaluation, while more recently published data have shown the feasibility of TAVI in intermediate and low-surgical risk patients as well. According to the 2021 European Society of Cardiology (ESC) guidelines, TAVI is recommended in patients who are at least 75 years of age or in those with high surgical risk evaluated by the Society of Thoracic Surgeons (STS) predicted risk of mortality (PROM) or a EuroSCORE II > 8%, as well as in those who are unsuitable for surgery [1]. These patients should generally be referred to TAVI (class I recommendation, level of evidence A) [1]. Patients who do not fall into the above-mentioned categories should be extensively and individually evaluated, considering not only clinical, anatomical, and procedural factors but also the individual risk-benefit ratio of both SAVR and TAVI procedures [1]. The vascular access evaluation is also very important, and, in most cases, femoral access is preferred. However, among others, trans-axillary, trans-carotid, or trans-apical routes could also be used, depending on anatomical features and risk factors [3]. Transthoracic echocardiography (TTE) is principal in the diagnosis of AS. By definition, severe AS is considered a state in which the aortic valve area (AVA) is less than 1.0 cm^2^ (0.6 cm^2^/m^2^ of body surface area [BSA]). Not only this parameter but also aortic valve pressure gradient, left ventricle ejection fraction (LVEF), and cardiac output determine the severity of stenosis and the type of AS. All the above-mentioned parameters help to determine the appropriate treatment algorithm [1,4]. Computed tomography (CT) is a preferred method to assess aortic valve, aorta, and iliac arteries anatomy and the level of their calcification. The TAVI procedure lasts a shorter time than the surgical method and is associated with a shorter length of hospitalization [5]. Additionally, TAVI, in comparison to cardiac surgery, significantly reduces the risk of major bleeding, especially life-threatening bleeding, acute kidney injury, and new-onset atrial fibrillation (NOAF) in the short postoperative period (30 days) as well as in the long-term follow-up (1 year) [6]. TAVI via the iliofemoral access sitemay also be supported by improvement in quality of life in the short-term postoperative period (1 month) compared to surgery, as expressed in self-assessment forms [7]. Thus, the TAVI procedure allows a faster discharge after the procedure and a faster improvement in the health status based on standardized questionnaires in the population of people > 75 years old and with frailty syndrome. The TAVI procedure, compared to a surgical operation, is much less invasive but has limitations. Contraindications for TAVI arise from factors limiting the procedural success and/or giving a high risk of complications, such as lack of vascular access, suboptimal or calcified iliac vessels or their tortuous course making the insertion of a vascular guide impossible or too risky, severe coexisting abnormalities of other heart valves, thrombus in the left ventricle, or active infective endocarditis [8,9]. Anatomical features such as a low coronary artery outflow (<12 mm) or small sinus of Valsalva (<30 mm in diameter) are also contraindications to TAVI [10]. Relative contraindications include untreated coronary artery disease requiring revascularization and hemodynamic instability [11,12]. In the case of relative contraindications, an important factor for evaluation is the individual clinical condition of the patient to determine the risk of abandonment and performing the procedure.

The aforementioned aspects are stated in the 2021 ESC guidelines regarding the treatment of valvular heart diseases, where the choice of TAVI is supported by a patient profile with higher surgical risk, older age (≥75 years), previous heart surgery (especially previous coronary artery bypass grafting and being at risk for the injury of intact grafts during sternotomy), severe frailty (defined as at least 2 factors in the Katz index), adequate transfemoral access, history of chest irradiation, porcelain aorta, high risk of patient-valve prosthesis mismatch (AVA < 0.65 cm^2^/m^2^ BSA), and severe thoracic deformity or scoliosis [1].

Therefore, when deciding on the treatment of AS, a comprehensive clinical assessment of the patient is crucial, considering the individual surgical risk and the existence of the associated factors. The above mentioned factors should be assessed by a qualified heart team and discussed in light of the center’s experience in treatment with both methods. The TAVI procedure is used successfully in patients at high surgical risk but may be also performed in patients younger than 75 years and with intermediate or low surgical risk [13,14,15,16]. Studies have shown no worse (non-inferiority) outcomes for patients with high and intermediate surgical risk undergoing TAVI compared to SAVR in a follow-up of up to 5 years [6,17,18,19,20,21,22,23,24,25,26,27]. A 5-year follow-up in intermediate-risk-for-SAVR patients undergoing TAVI showed that mortality or occurrence of a disabling stroke did not differ from the surgically treated group [25]. There are also data for no worse outcomes of TAVI in the low-surgical risk group of patients in a 2-year follow-up [28,29,30,31]. However, data on the benefits of TAVI in the low-risk-for-SAVR group are limited. The characteristics of these patients are different in comparison to patients who usually qualify for TAVI. One of the major problems is the durability of percutaneously implanted valves in younger patients [25]. Therefore, the potential risks and benefits of this method in younger patients should be deeply analyzed, especially with regard to potential complications after TAVI, noting that this procedure has a higher rate of vascular complications, perivalvular leaks, and cardiac conduction abnormalities, compared to SAVR [32].

## 2. Types of Cardiac Conduction Abnormalities after TAVI and Their Management

From the electrocardiological point of view, the most common complication after TAVI is the left bundle branch block (LBBB), occurring in 13.3–39% [33,34] of patients, but the most serious complication is an atrioventricular block (AVB) requiring implantation of a cardiac pacemaker (PM). These complications are related to the anatomical proximity of the atrioventricular (AV) junction and the aortic valve structures (the left bundle branch runs within the membranous septum directly under the tissue separating the non-coronary and right coronary leaflets [35]). Mechanical pressure on the AV node and the bundle of His structures by the expanding valve may cause conduction disturbances due to edema, local hematoma or ischemia of the conduction system structures [11,36].

The incidence of the need for PM implantation after TAVI is higher than for SAVR, ranging from 3.4% to 25.9% [37] or even 2% to 51% [38,39], depending on the study, while for SAVR it is 5.5% (relative risk [RR]: 2.43; 95% confidence interval [CI]: 1.62–3.63) [32]. Differences between reported rates of PM placement after TAVI are related to the most commonly used valve systems, with higher rates using the Medtronic CoreValve Revalving System compared to the Edwards Sapien Valve [39]. Time criteria for the onset of conduction disturbances allow a distinction to be made between early disturbances requiring PM occurring up to 48 h after the procedure and delayed disturbances defined as occurring over 48 h after the procedure (about 18% of conduction disturbances requiring PM after TAVI) [40]. The above percentages of need for PM regarding early and late disorders include all conduction disturbances requiring PM implantation. Regarding the incidence of LBBB, most manifest perioperatively or within 24 h of surgery; in the analysis by Auffret et al. [35], 85–94% of LBBB manifested perioperatively, whereas 6.6–17.8% occurred >24 h after surgery. In 55% of cases, the disturbance persisted for at least 30 days. Occurrences of LBBB after discharge to a 1-year follow-up are sporadic, with a frequency of 0–2.9% [35]. Thus, it seems that conduction disturbances that may suggest PM implantation stabilize 3 days after the procedure [41]. It has also been suggested that patients after TAVI can be discharged home within the first 48 h after the procedure if a balloon-expandable valve without predilatation was used or if they do not have factors for the development of delayed conduction disturbances, such as non-specific conduction disturbances with a QRS duration > 110 ms, a previous right bundle branch block (RBBB), the implantation of a self-expandable valve, and predilatation during the procedure because of the low risk of conduction disturbances after 48 initial hours [40]. However, if the patient has dynamic electrocardiographic (ECG) changes after the procedure, such as a new bundle branch block accompanied by prolongated PR or QRS intervals, then extended hospital monitoring of up to 5 days using ECG should be considered [37]. The nature of conduction abnormalities is an interesting issue. In the Placement of AoRtic TraNscathetER Valves (PARTNER) registry analysis by Nazif et al. at 1-year follow-up, 50% of patients with PM had pacing present on ECG evaluation, so the authors, in the absence of more detailed registry data on cardiac pacing dependence, suggested that pacing dependence in these patients is less than 50% [42]. In other analyzes [43], the observed cardiac pacing dependence was 16.1% after 30 days and 12.9% after 1 year. In another study by Costa et al. [44], pacing dependence was found in 35.7%, 35.8%, and 33.3% of patients at follow-ups of 1, 6, and 12 months, respectively. In the smallest of these studies, the assessed dependence at the 6- to 8-week follow-up after the procedure was 37% [45]. Analyses of the above data with decreasing pacing dependence over time may indicate the dynamic and, at least in some cases, the transient nature of the conduction disturbances due to the temporality of contributing factors, such as transient inflammation, edema, ischemia, and transient mechanical compression, which may resolve over time [44].

Less frequent potential indication for PM in patients after TAVI may be sinus sick syndrome (SSS) [46]. It is worth noticing that patients referred for TAVI are at an older age, frequently with comorbidities and structural heart diseases, which may predispose them to myocardial fibrosis, which is associated with both SSS and AV conduction abnormalities [47]. It was shown that SSS and AV conduction disturbances may coexist in up to 67% of patients and annually 0.6–1.9% of patients with SSS develop AVB, which suggests dual-chamber PM implantation when SSS is diagnosed [48,49].

Evaluation of related data shows that the occurrence of conduction disturbances after TAVI remains the most frequent complication of the procedure [6]. This consideration goes against the potential pressure toward a minimalist approach, including early discharge of TAVI recipients, and the possible risks associated with too-precipitous clinical decisions. Rodés-Cabau et al. [50] in the consensus regarding the management of conduction disturbances associated with TAVI formulated a multidisciplinary initial attempt to provide a guide based on the best available data and group expertise (interventional cardiologists, electrophysiologists, and cardiac surgeons) [50]. They proposed an interesting example for the evaluation of the patients, creating algorithm proposals for the management of patients related to the specific risk categories. According to this approach, patients with pre-existing RBBB, new-onset LBBB, high-degree AVB, complete AVB, or prior conduction disturbances and with new conduction changes reflected by PR or QRS interval prolongation (>20 ms) after the implantation procedure should be secured with temporary PM for 24 h. Patients with a persistent high-degree or complete AVB should be qualified for permanent PM. Careful re-evaluation is suggested in patients with a persistent increase of PR or QRS of at least 20 ms and pre-existing first-degree AVB or QRS duration ≥ 120 ms [50]. Electrophysiology study (EPS) or continuous ECG monitoring should be considered, according to the scientific expert panel, in cases of a further PR or QRS duration increase of at least 20 ms and in patients with a PR interval > 240 ms or a QRS duration > 150 ms in qualifying for permanent PM (rather not in the case of a PR interval > 240 ms, but a QRS duration < 120 ms) [50]. Highlighting the potentially transient nature of conduction disturbances after TAVI, some authors suggest performing EPS post-TAVI in order to decide about PM implantation in patients with equivocal indications. Rogers et al., in their study, showed that this strategy with positive EPS (intra/infrahisian block, His-Ventricle interval > 100 ms at baseline or after intravenous administration of 1 g of procainamide) as an indication for PM avoided implantation in 70% of dubious cases [51]. Postprocedural PR interval prolongation or cardiac axis change, after TAVI, may be caused by mechanical compression of the conduction system by a calcified aortic ring and/or by an implanted valve [52]. The 2021 ESC guidelines on cardiac pacing and cardiac resynchronization therapy [37] suggest several management pathways depending on preprocedural conduction abnormalities and post-procedural ECG findings. Their comparison with the American College of Cardiology (ACC)/American Heart Association Guidelines is shown in Table 1 [37,53].

What is also worth mentioning is that ESC guidelines state that PM should not be implanted as a prophylactic measure in patients with previous RBBB, who have no indications for cardiac pacing.

The perspective of possible conduction disturbances and lifetime pacing device presence should be considered. The necessity of PM replacement due to battery depletion and possible pacing-induced cardiomyopathy (PICM), along with pacing device-related complications, puts patients at risk of additional hospitalizations. Although studies show that PM after TAVI increases the risk of hospitalization and mortality in high-risk patients [54], data on younger individuals are still limited and results from trials on low-risk patients are needed [55].

Appropriate CIED selection for patients after TAVI is another issue in patients with coexisting heart failure with reduced ejection fraction (HFrEF). Generally, according to ESC 2021 guidelines, cardiac resynchronization therapy (CRT) is recommended in patients requiring ventricular pacing with high-degree AVB and HFrEF (LVEF < 40%) (class I recommendation, level of evidence A) or especially in symptomatic heart failure (HF) patients with sinus rhythm and LBBB QRS morphology (QRS duration ≥ 150 ms) [37,56]. Currently published data on CRT de novo implantation after TAVI and outcomes are limited; however, results are promising. Meguro et al. presented a case of a patient with new LBBB after TAVI and concomitant ischemic HFrEF during optimal medical treatment [57]. After TAVI, the patient was qualified for CRT-defibrillator (CRT-D) placement, which significantly decreased the patients' N-terminal pro–B-type natriuretic peptide levels and improved LVEF along with the left ventricular end-diastolic diameter [57]. Osmancik et al. described a case of primary dual-chamber PM implantation after TAVI with progressing HF symptoms and LVEF impairment due to right ventricular (RV) pacing despite optimal medical treatment. The patient was referred for an ‘upgrade’ to CRT afterward, which led to improvement in patient-reported symptoms and LVEF (increase from 25 to 45%) [58]. Decision-making on implanting PM or de novo CRT or introduction of conduction system pacing (CSP) with or without implantable cardioverter-defibrillator (ICD) capabilities after TAVI and choosing the right time for these procedures is challenging. Studies specifically addressing this issue would be valuable.

## 3. Factors Associated with Conduction Abnormalities after TAVI

In the assessment of complications after the TAVI procedure, the researchers aim to distinguish between factors influencing the occurrence of LBBB (as the most common complication) and factors predicting the need for PM implantation. Both situations may adversely affect patients’ subsequent prognosis [42].

In the PARTNER registry analysis, Nazif et al. showed that the need for PM implantation influences longer patient hospitalization, more frequent hospital readmissions, and higher mortality [42]. In the study by Dizon et al. [46], patients were divided into four groups based on having a PM after TAVI: patients with a previous PM, patients with a newly implanted PM, patients without a PM, and patients without a PM with a newly developed LBBB. With newly implanted PMs, the most frequent indications were respectively: AVB (79%) and SSS (17.3%). For implantable devices, the majority of indications were dual-chamber PM (75.7%), single-chamber PM (19.7%), biventricular PM (CRT-P, 2.9%), ICD (0.6%), and CRT-D (0.6%). Data analysis showed higher mortality in each patient group compared to the group without implanted PM. Newly implanted PM and prior PM influenced increased mortality after one year of follow-up as well as reduced LVEF after the same follow-up time compared to the group without PM [46]. This supports the view on the worse prognosis of patients with implanted PM after the TAVI procedure. Also, in patients with chronic LBBB and no PM after TAVI, LVEF is reduced compared to patients without LBBB and no PM after 1 year of follow-up (48.9 ± 11.2% vs. 57.6 ± 8.2%, respectively; *p* < 0.0001) [46]. The presence of new or prior PM was an independent predictor of mortality after TAVI, similar to liver failure, atrial fibrillation (AF), anemia, renal disease, male gender, chronic obstructive pulmonary disease, and STS score (hazard ratio [HR]: 1.31; 95% CI: 1.08–1.60; *p* = 0.006, for previous PM compared to no PM.; HR 1.38 [95% CI: 1.00–1.89], *p* = 0.05, for new PM compared to no PM).

These observations are in line with data from the American STS/ACC registry analysis [54]: PM implantation was associated with longer hospital stay (7 days vs. 6 days; *p* < 0.001) and intensive care unit stay (56.7 h vs. 45.0 h; *p* < 0.001). PM implantation was also associated with increased mortality (24.1% vs. 19.6%; HR: 1.31; 95% CI: 1.09–1.58) and a composite endpoint including death or hospitalization for HF (37.3% vs. 28.5%; HR: 1.33; 95% CI: 1.13–1.56) at 1 year, but not with HF admission alone (16.5% vs. 12.9%; HR: 1.23; 95% CI: 0.92–1.63) [54]. However, the strength of the above conclusions was contrasted with other studies [59,60,61,62] and two large meta-analyses. Reguiero et al. [33] demonstrated no difference in 1-year follow-up mortality after PM implantation (RR: 1.03; 95% CI: 0.9–1.18), and even a protective effect of PM against cardiac death was observed (RR: 0.78; 95% CI: 0.60–1.03). Mohananey et al. [63], in a study in which researchers pooled over 20,000 patients from 23 studies, found no significant difference in both 30-day and 1-year overall mortality, cardiovascular mortality, myocardial infarction, and stroke. A significantly different factor was the greater improvement in LVEF in patients without implanted PM compared to patients with PM (standardized mean difference: 0.22; 95% CI: 0.12–0.32). The authors stated that the lower proportion of implanted PMs in the study group and the significant and disproportionate loss of patients with implanted PM at the 1-year follow-up compared to patients without PM is responsible for the difference in results compared with the STS/ACC registry analysis by Fadahunsi et al. (loss to follow-up 76% vs. 36%). However, researchers agree on the deterioration of left ventricular function in the group of patients with implanted PM [33,34,54].

Studies attempting to describe risk factors for new LBBB highlight two main groups of predictors: anatomical and procedural. The anatomical factors include a massive amount of calcifications on the aortic valve and accumulation of calcifications in the left coronary leaflet area of the aortic valve (patients with calcium load of left coronary cusp above 209 mm^3^ had a greater risk of PM implantation) [12], a shorter membranous septum, and an eccentric left ventricular outflow tract (LVOT) [64]. Procedural factors include valve implantation at the level of the non-coronary leaflet, a valve diameter too large in relation to the aortic annulus (>15% [oversizing]), the use of the Medtronic CoreValve compared to the Edwards Sapien system, deeper implantation, the stretching of the aortic annulus, and a larger valve diameter with a suggested 26 mm for Medtronic and 29 mm for Edwards Sapien valves [35]. In their review paper, Auffret et al. [35] summarized the main predictors of new LBBB occurrence from published studies with multivariable analyses. These include Medtronic CoreValve implantation compared to Edwards Sapien (odds ratio [OR]: 2.5–8.5), deeper implantation (per 1 mm OR: 1.15–1.4), significant dilatation of the native aortic valve annulus by the prosthesis (OR: 1.8 per 1%; 5.3 if >15% dilatation of native aortic valve annulus), larger valve diameter—for Medtronic CoreValve 26 vs. 23 mm (OR: 4.1) for Edwards Sapien 29 vs. 20–23 mm (OR: 3.12). Other factors may also have an impact, such as previous conduction disturbances (especially prolonged QRS), female sex, previous coronary artery bypass grafting, diabetes, and aortic valve calcification [35].

The meta-analysis by Siontis et al. [39] covering 41 studies and 11,210 patients identified the following factors associated with elevated risk of PM placement: male sex, first-degree AVB, left anterior hemiblock (LAH), RBBB, and periprocedural AVB. Regardless of the type of valve used, the authors concluded that the use of Medtronic systems increases the risk of PM implantation by 2.5 times compared to Edwards Sapien (RR: 2.54; 95% CI: 2.08–3.12). Interestingly, some authors report that balloon-expandable valve implantation without predilatation did not affect the occurrence of conduction disturbances that would manifest after 48 h [40]. It was suggested that self-expandable valves probably need more time to position themselves in the aorta; therefore, these valves would be responsible for conduction disturbances after 48 h [40]. Similar conclusions were drawn from a meta-analysis of the American Thoracic Society Registry and the ACC [53].

**Figure 1:**
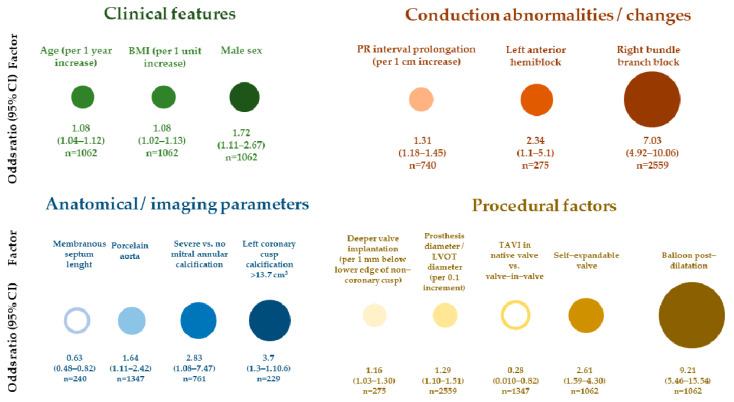
Main factors associated with permanent pacemaker implantation after transcatheter aortic valve implantation. Shown odds ratios and their magnitudes (reflected by the sizes of the circles within subgroups of factors) result from multivariable analyses of major clinical studies. Based on: [37,42,52,65,66,67,68,69,70]. BMI: body mass index, CI: confidence interval, LVOT: left ventricular outflow tract, n: number of studied patients.

Factors associated with the requirement of PM implantation after TAVI can be divided into clinical, anatomical/imaging parameters, conduction abnormalities (with emphasis on pre-existing conduction disturbances), and periprocedural factors, depending, among others, on the diameter of the implanted valve, the method of implantation, and, the course, of the procedure itself (Figure 1).

Auffret et al. [35], in their review of the literature considering 48 studies with multivariate analysis, list the main factors for the need for PM implantation after TAVI. These are (ranges from other studies) previous RBBB (OR: 2.8–46.7), Medtronic CoreValve implantation (OR: 2.6–25.7), implantation depth (OR: 1.1–1.5/per 1 mm increment), oversizing/stretching of the aortic ring/LVOT (OR: 1.02–1.5/per 1% increment), and first-degree AVB (OR: 4.0–11.4). Analyzing the above data, one can find different investigators listing similar predictive factors of PM, but Auffret et al. [35] point out that possibly one of the strongest and most common predictor of PM is a prior history of RBBB. Along with this, the authors point out that new LBBB, calcification of the aortic valve, LVOT and mitral annulus, and implantation depth are also important. The proposed implantation depth cutoff values associated with new LBBB or permanent PM placement are 5–6.7 mm for Lotus valve, 6.3 mm for Edwards Sapien XT, 6–7.8 mm for CoreValve system, and 7 mm for Edwards Sapien 3 [35]. Also, the 2021 ESC pacing guidelines emphasize the earlier occurrence of RBBB as very significant factor influencing the need for cardiac pacing after TAVI [37]. The main factors predisposing to permanent PM implantation after TAVI are shown in Figure 1 and Table 2 [35].

When analyzing risk factors for PM implantation within 30 days, predispositions to PM were age, previous conduction disturbances, aortic valve area when ≤0.75 cm^2^, procedural risk classification, type of implanted valve, and access site (Table 2). The authors also detailed negative factors: previous aortic valve surgery, use of home oxygen therapy, and longer procedure time (per 30-min increment) [54]. Also, a lower risk was observed among inoperable/extreme surgical risk patients and patients on home oxygen therapy (Table 2). The authors explained, at least in part, such a phenomenon of the reduced rate of need for PM implantation in these patients by potentially less oversizing caused by operators who are more mindful due to the disease burden of such patients [54]. A summary of factors associated with PM implantation after TAVI based on the two studies involving significant numbers of patients is included in Table 2 [39,54], while the TAVI systems with PM implantation rates are summarized in Table 3 [71,72].

Moskowitz et al., on the other hand, analyzed factors predisposing for permanent PM implantation after SAVR, showing that older age and prior conduction/heart rhythm disturbances (fascicular block, high-degree AVB, and sino-atrial node dysfunction) were similarly predisposing for PM placement, as in TAVI patients [73].

Interesting observations with reference to the above data are presented in the study by Mauri et al. [68] analyzing the risk of PM using Edwards Sapien 3 Transcatheter Heart Valves alone. The researchers suggested that apart from the independent risk factors mentioned earlier (previous RBBB, significant LVOT calcification at the level of the right and left coronary leaflets, and deeper valve implantation), the main factor that may influence the need for PM implantation after TAVI is the operator’s technique. A reduction in the percentage of PM may be achieved by modification of the valve implantation technique, e.g., higher valve implantation by 3 mm results in a 52% reduction in the need for PM without an increase in perivalvular leak. Higher valve implantation could be achieved by cautious and optimized implantation using a novel technique called cusp overlap. The method aims to provide effective implantation of self-expandable valves by overlapping the view of the right and left coronary cusp in fluoroscopy [74]. Results of using this technique are promising, suggesting that it may reduce the risk of PM implantation after TAVI, compared with traditional 3-cusp coplanar projection, from 21.7% to 11.8% (*p* = 0.03), due to significantly higher new valve implantation [75]. On the other hand, operators should be aware of the possible risk of valve embolization and aortic regurgitation when the valve is deployed too high [76].

**Figure 2:**
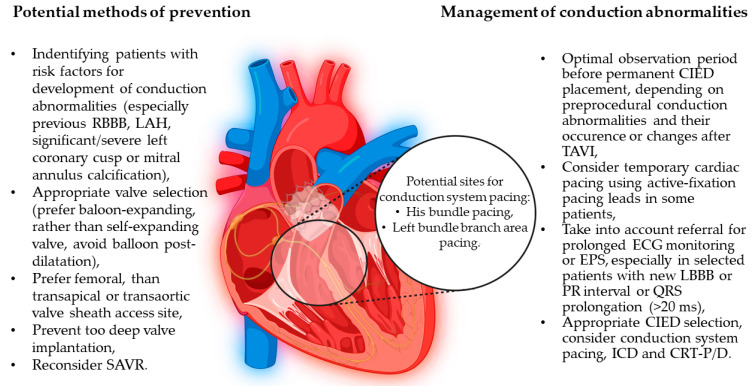
Prevention and management of conduction abnormalities after transcatheter aortic valve implantation. A schematic image of anatomical relationships of the conduction system structures in the aortic valve area. CIED: cardiac implantable device, CRT-P/D: cardiac resynchronization therapy-pacemaker/defibrillator, ECG: electrocardiogram, EPS: electrophysiology study, ICD: implantable cardioverter-defibrillator, LAH: left anterior hemiblock, RBBB: right bundle branch block, LBBB: left bundle branch block, SAVR: surgical aortic valve replacement, TAVI: transcatheter aortic valve implantation.

Thus, it is suggested that a meticulous analysis of patient characteristics, including the presence of previous ECG conduction abnormalities (RBBB) and the presence of calcifications at the LVOT level, is crucial when attempting to reduce the risk of PM placement. Pre-procedural CT could have the benefit of identifying patients with more massive calcifications in order to modify the procedure in them for higher valve implantation [64]. Also, careful selection of the implanted valve could be important, as oversizing as well as larger diameter implanted valves may influence the occurrence of new LBBB. Additionally, the use of implantable electrocardiographic monitoring devices in patients with new LBBB seems worthy of consideration. Data collected from the Ambulatory Electrocardiographic Monitoring for the Detection of High-Degree Atrio-Ventricular Block in Patients With New-onset PeRsistent LEft Bundle Branch Block After Transcatheter Aortic Valve Implantation (MARE) study postulate the relevance of implantable loop recorder (ILR) implantation in patients with new-onset LBBB persisting >3 days after TAVI, which may change the therapy in patients undergoing such monitoring [77] (Figure 2). Results of ILR interrogation in 12-month follow-up, revealing high-degree AVB, severe bradycardia, or ventricular arrhythmia, led to CIED implantation in 11% of observed patients [77].

The patient’s cardiac anatomy affecting the risk of possible conduction blocks relates mainly to variation in the length of the membranous septum, variation in the location of the AV junction, and the spatial distribution and degree of calcification on the aortic valve leaflets. The personal variability regarding the location of the AV junction and the resulting susceptibility to conduction disturbances after TAVI was described by Kawashima and Sato [38]. In their study, they examined 115 autopsy specimens, observing the course of the AV junction on the interventricular septum (IVS). In 50% of cases, the conduction structures were located on the right side of the IVS, and in 30% of patients, they were on the left. In 20% of patients, the AV junction was located shallowly, subendocardially, at the very top of the IVS. The latter two variants were associated with higher rates of conduction disturbances in the form of LBBB and AVB. However, these are microanatomical factors currently impossible to evaluate in preoperative studies. An interesting group of studies is publications that consider measurements of cardiac anatomical structures that are feasible on preprocedural CT, thus providing an opportunity to develop tools to objectively assess the risk of PM placement after TAVI. The membranous septum is the upper part of the IVS, extending from the muscular septum upwards and to the right to the fibrous ring of the aorta. For electroanatomical reasons, it is an important reference point due to the course of the so-called penetrating part of the His bundle, which pierces the central fibrous body to emerge finally at the top of the muscular septum, being somehow squeezed between the membranous and muscular components of the IVS [11]. The length of the membranous septum correlates with the distance of the aortic valve annulus from the AV junction, and may, therefore, be an important and relatively simple parameter to assess on preoperative CT imaging to predict conduction disturbances after TAVI [11,69]. A relationship between the length of the membranous septum measured on preoperative CT and the occurrence of conduction disturbances requiring PM was demonstrated by Maeno et al. (Figure 1), who also proposed a parameter taking into account the difference between membranous septum length and aortic valve implantation depth [69].

The distribution and degree of calcification of the aortic valve leaflets and mitral annulus appear to be another important and quantifiable parameter useful in the prognosis after TAVI implantation [12,78,79]. Fujita et al. demonstrated an increased incidence of PM implantation in patients with a calcification distribution predominant on the left coronary leaflet of the aortic valve, reporting as high as 53.9% probability of PM implantation with a corresponding degree of left coronary cusp (LCC) calcification and coexisting RBBB [12]. Authors suggested that LCC calcification causes a shift of implanted valves towards right- and non-coronary cusps, which predisposes to damage of conduction system structures in the nearby [12]. Parameter which is also assessable on preoperative multidetector CT (MDCT) and appears to be helpful in estimating the risk of conduction disturbances and consequent PM after TAVI is LVOT eccentricity (defined as 1-[minimal diameter/maximal diameter] × 100%) [64]. This is a parameter indicating LVOT geometry—approximately circular in the case of low eccentricity and more ellipsoid in the case of increased eccentricity [64]. A value >35% of LVOT eccentricity is postulated by the authors as an independent risk factor for new LBBB after Evolut 3 valve implantation. The authors also presented an interesting conclusion—when detecting high risk anatomy, membranous septum length was less than 6.5 mm and/or LVOT eccentricity >35%, they suggest considering modification of procedural factors—decreased valve implantation depth and less valve oversizing to reduce the risk of new LBBB.

## 4. Pacing-Induced Cardiomyopathy and Potential Solutions

Assessment of the risk of PM implantation is particularly important due to the studies based on the PARTNER III registry proposing TAVI also in low- and intermediate-risk patients. Implantation of PM, especially when a high percentage of RV pacing is expected, is defined as at least 20% of RV pacing, which was found to be related to the development of PICM [80] and is associated with poor outcomes. As documented, e.g., in the Dual Chamber and VVI Implantable Defibrillator (DAVID) study [81], in a cohort of HF patients with dual-chamber ICD placed, where dual-chamber rate-responsive pacing was 70/min compared to VVI backup pacing of 40/min, showed a higher risk of a composite endpoint of hospitalization for congestive HF and mortality. Similar conclusions were drawn from the Biventricular versus Right Ventricular Pacing in Heart Failure Patients with Atrioventricular Block (BLOCK HF) study [82], which has shown fewer hospitalizations and a reduction in mortality in patients with AVBs and LVEF up to 50% who received biventricular vs. RV pacing. Myocardial electrical and mechanical dyssynchrony are thought to be the causes of the above outcomes [83] and result from deterioration of both left ventricular systolic and diastolic function. The effect of a shortened diastolic phase on poorer coronary perfusion of the myocardium has also been suggested [83,84]. This may lead to myocardial remodeling and the development of PICM [85], defined as a decrease in LVEF in patients with high percentages of RV pacing. PICM according to some studies may affect approximately 10–20% of patients 3–4 years after PM placement [86]. Importantly, an analysis of the PARTNER trial registry performed by Dizon et al. [46] demonstrated reduced LVEF in patients undergoing complicated TAVI requiring PM implantation after one year, while patients with a previously implanted PM showed poorer recovery of LVEF after valve implantation. The results of the above studies suggest an important clinical problem—TAVI in younger patients is associated with a higher risk of developing adverse effects of RV pacing if PM is required, due to the longer pacing period. Therefore, assessment of the risk of conduction disturbances requiring PM, as well as definition of risk factors for PM, especially in low- and intermediate-risk patients, is reasonable, taking into account that SAVR is associated with a significantly lower risk of conduction disturbances requiring PM implantation (14.4% vs. 5.5%) [32].

**Figure 3:**
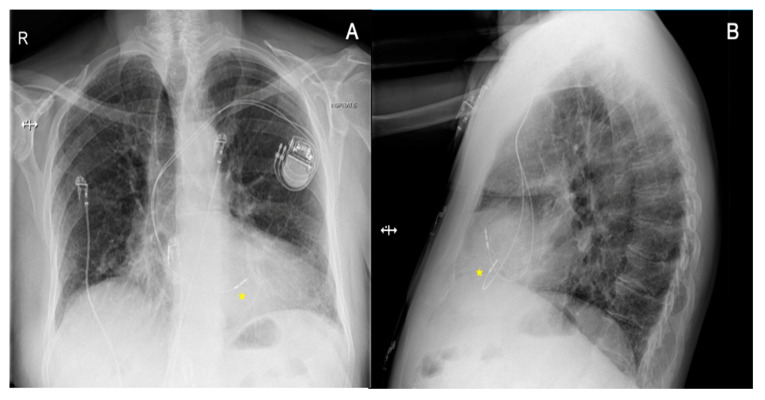
Chest X-rays of a patient after transcatheter aortic valve implantation who developed a complete atrioventricular block and received a pacemaker with conduction system pacing. A posterior-anterior view (panel **A**) and a lateral view (panel **B**). An atrial lead was placed in the right atrial appendage, while a ventricular lead was placed in the left bundle branch area pacing site (indicated by asterisk).

**Figure 4:**
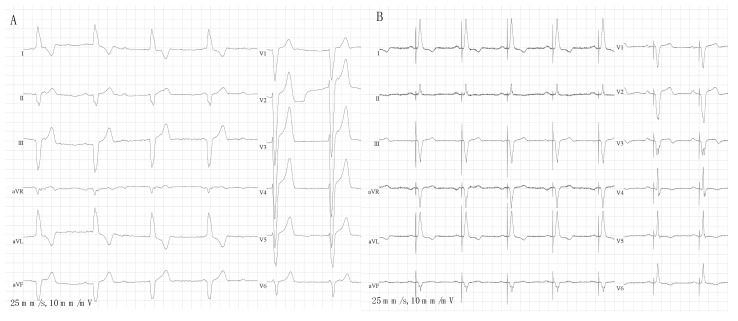
Twelve-lead ECGs in a patient after transcatheter aortic valve implantation with a complete atrioventricular block (panel **A**) who subsequently underwent implantation of permanent pacemaker with left bundle branch area pacing (panel **B**). Left ventricular activation time during pacing was 77 ms.

There is also growing evidence that new methods of cardiac pacing, such as CSP may be a feasible alternative to RV pacing in order to mitigate its potential consequence of LVEF deterioration [87,88]. CSP involves targeting His bundle pacing (HBP) or left bundle branch area pacing (LBBAP), which aims to result in more physiological electrical impulse propagation and less ventricular dyssynchrony [87,88,89]. CSP is emerging as a valid therapeutic strategy in patients with conduction system diseases aiming to restore the physiological electrical activation of the ventricle(s). It offers a rational for mechanical dyssynchrony correction, especially in patients with decreased LVEF and in patients at high risk of PICM [90,91,92,93]. Vijayaraman et al. [92] reported a retrospective analysis of data on physiological pacing, including HBP and LBBAP, in patients who required permanent device implantation following TAVI: His-Purkinje CSP was successful in 55 of 65 post-TAVI patients (85%), with a mean age of 79 ± 8 years. HBP was successful in 29 of 46 patients (63%), while LBBAP was successful in 26 of 28 (93%). The mean follow-up period for the entire cohort was 12 ± 13 months (median: 8 months). No differences were observed in LVEF and left ventricular end-diastolic diameter during follow-up. The authors highlighted that, during the procedure, the valve was used as a fluoroscopic marker for His bundle region localization. There is a need for large studies to evaluate the long-term outcomes of these techniques. However, currently available results on CSP, including in patients after TAVI, are promising [94]. Chest X-rays and ECG findings before and after the procedure in a patient who, due to severe AS, was referred for TAVI (Portico Valve) and subsequently developed complete AVB and received permanent PM are shown in Figure 3 and Figure 4. The patient before TAVI had first-degree AVB.

## 5. Other Heart Rhythm Disorders after TAVI

Patients with AS undergoing TAVI and SAVR, in addition to conduction disturbances, especially in the form of LBBB or AVB, frequently present with AF or NOAF, which is a significant problem. An association between the pathophysiology of AF development and AS can be noted, as both conditions have similar risk factors, such as hypertension, age, obesity, the presence of sleep apnea, and increased pericardial fat [95]. Available data suggest that concomitant AF is more common in patients undergoing TAVI than SAVR (32.1% vs. 12.8%, respectively), which may be related to the older age of the patients and comorbidities [96,97]. However, NOAF occurs more frequently after SAVR than after TAVI (30.7% vs. 3.5%, respectively TAVI), where the influence of procedural factors related to the surgery, including pericardiotomy, aortic clipping, and the use of extracorporeal circulation, have been postulated [96,97]. A meta-analysis by Sannino et al. [98] reports that in patients undergoing TAVI, prior AF occurred in 33.4% and NOAF in 17.5% of cases, supporting the view that TAVI patients are more likely to have prior AF than NOAF. An observational population-based study utilizing International Classification of Diseases, Ninth Revision (ICD-9) codes from a national registry covering 171,480 hospitalizations of patients undergoing aortic valve replacement showed that NOAF was observed in 50.4% of hospitalizations of TAVI patients and 50.1% of patients who underwent SAVR [99]. NOAF in TAVI was more often associated with the use of transapical access compared to the transfemoral route, whereas in SAVR, it was associated with biological valve implantation compared to mechanical [99]. However, the researchers in the above paper highlight the limitations of conducting a large observational study based on the analysis of ICD-9 codes, which may have influenced the overestimation of NOAF and problems with discrimination between prior AF and NOAF. An analysis of another large registry, including 72,660 patients undergoing TAVI with non-apical access, showed the coexistence of AF in 40.7% of patients before the procedure, while the NOAF rate was 6.8% [100]. These data support the existence of an association between the presence of surgically treated AS and AF, which may affect patients’ subsequent prognosis. Furthermore, NOAF was associated with a higher risk of bleeding, stroke, and HF compared with prior AF [100]. Also, prior AF was associated with poorer prognosis and increased mortality compared to patients without AF (HR: 1.53; 95% CI: 1.47–1.58) [100]. Biviano et al. report that the presence of AF at baseline and discharge of a TAVI patient may be a factor for increased 1-year mortality (adjusted HR: 1.88; 95% CI: 1.50–2.36), while conversion from sinus rhythm to AF may be associated with increased 30-day and 1-year mortality (adjusted HR: 3.41, 95% CI: 1.78–6.54 and adjusted HR: 2.14, 95% CI: 1.45–3.10, respectively). Statistical models included, among others, the presence of bleeding requiring transfusion, renal failure requiring dialysis, and stroke, all defined in the PARTNER trial protocol [19,101].

These data differ from the meta-analysis by Sannino et al. [98], which similarly found that earlier AF, compared to sinus rhythm, was associated with increased mortality (HR = 1.68; 95% CI: 1.45–1.96) in the long-term follow-up (from 6 months to 5 years of observation after the procedure), but showed similar 30-day mortality in patients with sinus rhythm and earlier AF and no significant difference between short- and long-term mortality in patients with sinus rhythm and in those who developed NOAF. The impact of NOAF on post-TAVI complications and patients' prognosis appears inconclusive. According to the investigators, there are difficulties in identifying and distinguishing between patients with pre-existing AF and NOAF, as well as too low power of the studies performed to assess possible complications associated with NOAF after TAVI [100]. Factors such as oxidative stress or increased inflammatory response after the procedure, as well as left ventricular overload, may be responsible for the possible worsening of prognosis in NOAF [102,103]. It can be concluded that the influence of prior AF on the worse prognosis of patients after TAVI compared to those with preserved sinus rhythm seems to be more established. It has been postulated that long-term mortality, in this case, is influenced by thromboembolic complications or worsening of HF due to the presence of tachyarrhythmic cardiomyopathy and/or reduced cardiac output [104,105,106,107,108,109].

In addition to AF being the most common arrhythmia related to TAVI, other arrhythmias including supraventricular (SA) and ventricular arrhythmias (VA) were reported [110]. In a meta-analysis by Siontis et al. reviewing 43 506 patients from 65 studies, detection of new-onset sustained SA or VA was reported in 29 (16%) and 28 (3%) patients, respectively. However, these numbers may be underestimated due to the non-specified category of “major arrhythmia during the procedure” used by researchers, which occurred in 846 (30%) patients and could comprise both SA and VA [110].

VA is observed in patients with severe AS, and TAVI was found to have a protective impact on their occurrence. Tempio et al. assessed the prevalence of VA according to a modified Lown grading system in patients after TAVI and have shown a reduction in the frequency and severity of VA [111]. Reduction of the VA burden was observed as early as 1 month after TAVI. Importantly, the incidence of complex premature ventricular contractions (defined as grade > 2 of the Lown grading system) decreased from 48.6% to 16.4% of the patients 1 year after the procedure [111].

Also, cases of specific VA, such as bundle branch reentrant ventricular tachycardia, after TAVI have been reported. It was suggested that observed atrioventricular conduction delay after TAVI can create conditions (long HV interval) predisposing to this type of arrhythmia. Authors suggest that deep valve deployment causes damage via a calcified aortic valve on His-Purkinje fibers and enables a re-entry loop between the right and left bundle of His [112].

## 6. Conclusions

Optimizing the treatment of patients with severe AS can be achieved from the introduction of the above-mentioned clues into clinical practice. Careful patient selection for either TAVI or SAVR has a significant impact on a patient's prognosis. TAVI procedures performed in younger patients and in those at low or average surgical risk should prompt more detailed consideration of the probability of possible TAVI complications, and among them, newly occurring conduction abnormalities, including complete or high-degree AVBs requiring various CIED placement. The presence of significant conduction abnormalities after TAVI is associated with a worse prognosis; therefore, accurate assessment of the patients, before the procedure, including the evaluation of clinical, ECG, and anatomical factors, should be an important step in the planning and performing of TAVI procedures to reduce the risk of their occurrence. In patients with new or exaggerated conduction abnormalities after TAVI, appropriate diagnostics and treatment of conduction disturbances should be introduced, taking into account possible consequences of these treatment modalities in the follow-up. Accumulating evidence suggests consideration of CSP in patients requiring permanent cardiac pacing after TAVI.

## Figures and Tables

**Table 1:** Overview of the European Society of Cardiology and American College of Cardiology/American Heart Association Guidelines regarding cardiac conduction abnormalities after TAVI management.

Management	ESC 2021 Guidelines	ACC/AHA 2018 Guidelines
PM implantation	PM implantation: high- or third-degree AVB persisting for 24–48 h or new alternating BBB after TAVI procedure (class I recommendation, levels of evidence B or C, respectively), PM implantation within 24 h or immediately after TAVI: previous RBBB with new conduction abnormalities (change in QRS axis, increase in PR interval, transient high-degree AVB) (class IIa recommendation, level of evidence B).	PM implantation before discharge: new AVB with symptoms/hemodynamic instability (class I recommendation, level of evidence B) PM implantation: new persistent LBBB (class IIb recommendation, level of evidence B)
Additional examination/observation	Continuous ambulatory ECG monitoring for 7–30 days or EPS ≥ 3 days after TAVI: New LBBB and QRS duration > 150 ms or PR > 240 ms without change in duration at >48 h after the procedure (class IIa recommendation, level of evidence C) * QRS or PR increase > 20 ms with previous conduction abnormalities without further prolongation during 48-h observation (class IIb recommendation, level of evidence C)	Careful observation: new persistent BBB (class II b recommendation, level of evidence B)

ACC/AHA: American College of Cardiology/American Heart Association, AVB: atrioventricular block, BBB: bundle branch block, ESC: European Society of Cardiology, LBBB: left bundle branch block, RBBB: right bundle branch block, PM: pacemaker. * High-risk features for high-degree AVB development in patients with new LBBB consist of: LVEF < 40%, atrial fibrillation and prolonged PR interval.

**Table 2:** Selected clinical, electrocardiographic, anatomical, and procedural factors associated with pacemaker implantation after transcatheter aortic valve implantation.

Siontis et al. [39] a Meta-Analysis, n = 11,210, 17% Required PM RR (95% CI)	Fadahunsi et al. [54] n = 9785, 6.7% Required PM within 30 Days of TAVI OR (95% CI) *
Male sex: 1.23 (1.10–1.38)	Age (per 5-year): 1.07 (1.01–1.15)
First-degree AVB: 1.52 (1.15–2.01)	Previous conduction defect: 1.93 (1.63–2.29)
LAH: 1.62 (1.17–2.25)	Aortic valve area when ≤0.75 cm^2^ (per 0.25 cm^2^): 1.21 (1.00–1.45)
Right bundle branch block: 2.89 (2.36–3.54)	Self-expanding MCRS vs. balloon-expanding ESV: 7.56 (5.98–9.56)
Intraoperative AVB: 3.49 (2.49–4.89)	Access site (for valve sheath): transapical vs. femoral 1.36 (1.10–1.68) transaortic vs. femoral 1.52 (1.09–2.11)
MCRS vs. ESV: 2.54 (2.08–3.12)	Surgical risk (STS PROM): high vs. inoperable/extreme 1.85 (1.54–2.21) intermediate vs. inoperable/extreme 1.78 (1.04–3.04)
	Home oxygen use: 0.67 (0.49–0.91)
	Previous aortic valve procedure: 0.74 (0.57–0.95)
	Procedure time (per 30 min): 0.95 (0.92–0.99)

AVB: atrioventricular block, ESV: Edwards SAPIEN Valve, LAH: left anterior hemiblock, MCRS: Medtronic CoreValve Revalving System, STS PROM: Society of Thoracic Surgeons Predicted Risk of Operative Mortality. * Other predictors included in the model: chronic lung disease, contrast volume, hypertension, post-procedural mean aortic valve gradient, prior other cardiac surgery, prior percutaneous coronary intervention, procedure location, sex, and valve oversizing.

**Table 3:** Transcatheter aortic valve implantation systems with pacemaker rates. Based on: [71,72].

PM Implantation Rate after 30 Days or after 1 Year	Name of the Valve and Manufacturer	Type of TAVI System	PM Implantation Rate after 30 Days or after 1 Year
Low risk (<10%)	Acurate (Boston Scientific)	self-expanding	2.5%
	J-valve (Jie Cheng Medical Technologies)		2.3–4.8% **
	Acurate neo™ (Boston Scientific)		6.7%
	NEO 2 (Boston Scientific)		6.0–7.7%
	Myval (Meril Life Sciences)	balloon-expandable	5.8–8%
	Hydra (Vascular Innovations)	self-expanding	7.5%
Low-moderate risk (5–20%)	Portico (Abbott)	self-expanding	9.7% or 8.8–15.8% *
	Sapien 3 (Edwards Lifesciences)	balloon-expandable	8.2–10.1%
	Venus-A (Venus Medtech)	self-expanding	7.4–18.8%
	CoreValve Evolut R (Medtronic)		8.3–11.7%
	Evolut Pro (Medtronic)		10.8–11.9%
	JenaValve (JenaValve)		12.1%/9.1% *
Moderate risk (10–20%)	Navitor (Abbott)	self-expanding	15%
	Direct Flow Medical (Direct Flow Medical)	mechanical	13–17%
	wVR VitaFlow (Microport)	self-expanding	16.4–19.1% **
High risk (>20%)	Centera (Edwards Lifesciences)	self-expanding	27%
	Engager (Medtronic)		28.5%
	Lotus™ Valve (Boston Scientific)	mechanical	23.4–28.6%

* Data based on [72]. ** Indicates 1-year PM implantation rate. PM: pacemaker, TAVI: transcatheter aortic valve implantation.

## Data Availability

Not applicable.
